# A improved pooling method for convolutional neural networks

**DOI:** 10.1038/s41598-024-51258-6

**Published:** 2024-01-18

**Authors:** Lei Zhao, Zhonglin Zhang

**Affiliations:** School of Electronics and Information Engineering, Lanzhou Jiaotong University, Lanzhou, 730070 China

**Keywords:** Computational science, Computer science

## Abstract

The pooling layer in convolutional neural networks plays a crucial role in reducing spatial dimensions, and improving computational efficiency. However, standard pooling operations such as max pooling or average pooling are not suitable for all applications and data types. Therefore, developing custom pooling layers that can adaptively learn and extract relevant features from specific datasets is of great significance. In this paper, we propose a novel approach to design and implement customizable pooling layers to enhance feature extraction capabilities in CNNs. The proposed T-Max-Avg pooling layer incorporates a threshold parameter T, which selects the K highest interacting pixels as specified, allowing it to control whether the output features of the input data are based on the maximum values or weighted averages. By learning the optimal pooling strategy during training, our custom pooling layer can effectively capture and represent discriminative information in the input data, thereby improving classification performance. Experimental results show that the proposed T-Max-Avg pooling layer achieves good performance on three different datasets. When compared to LeNet-5 model with average pooling, max pooling, and Avg-TopK methods, the T-Max-Avg pooling method achieves the highest accuracy on CIFAR-10, CIFAR-100, and MNIST datasets.

## Introduction

Machine learning^1^ is one of the most exciting research directions in today’s science and technology field. As a core component of artificial intelligence, machine learning aims to develop algorithms and models that enable computer systems to learn automatically from data and make accurate predictions and intelligent decisions based on the learned knowledge. Over the past few decades, machine learning has made remarkable breakthroughs and has been applied in various fields, including image recognition^2^, natural language processing^3^, medical diagnosis^4^^5^^6^, financial forecasting^7^, etc.

The principles of machine learning can be traced back to the research on artificial intelligence in the 1950s. However, with the enhancement of computational power and the availability of large-scale data, machine learning has developed rapidly and has given rise to many important theories and methods. Among them, deep learning, as a branch of machine learning, has rapidly emerged and achieved significant breakthroughs in multiple domains in the past decade. Deep learning constructs multi-layer neural network models that can efficiently learn complex feature representations, thus achieving excellent performance in areas such as image classification^8^, speech^9^, and natural language.

Deep learning^10^ is a branch of machine learning that leverages multi-layer neural network models to efficiently learn and represent complex data. In recent years, deep learning has made revolutionary breakthroughs in fields such as computer vision, natural language processing, and speech recognition, surpassing traditional machine learning approaches in many tasks. Its notable advantage lies in its ability to automatically learn feature representations from large-scale data and handle non-linear relationships, extract abstract features, and process large-scale data. One of the key aspects of deep learning networks is convolutional neural networks^11^ (CNNs). CNNs consist of convolutional layers, activation layers, pooling layers, and batch normalization layers, among others. They are designed to extract features from raw data^12^, specifically in the domain of image analysis. The rapid progress in computer technology and hardware in recent years has provided support for building deeper networks and solving various problems.

**Figure 1:**
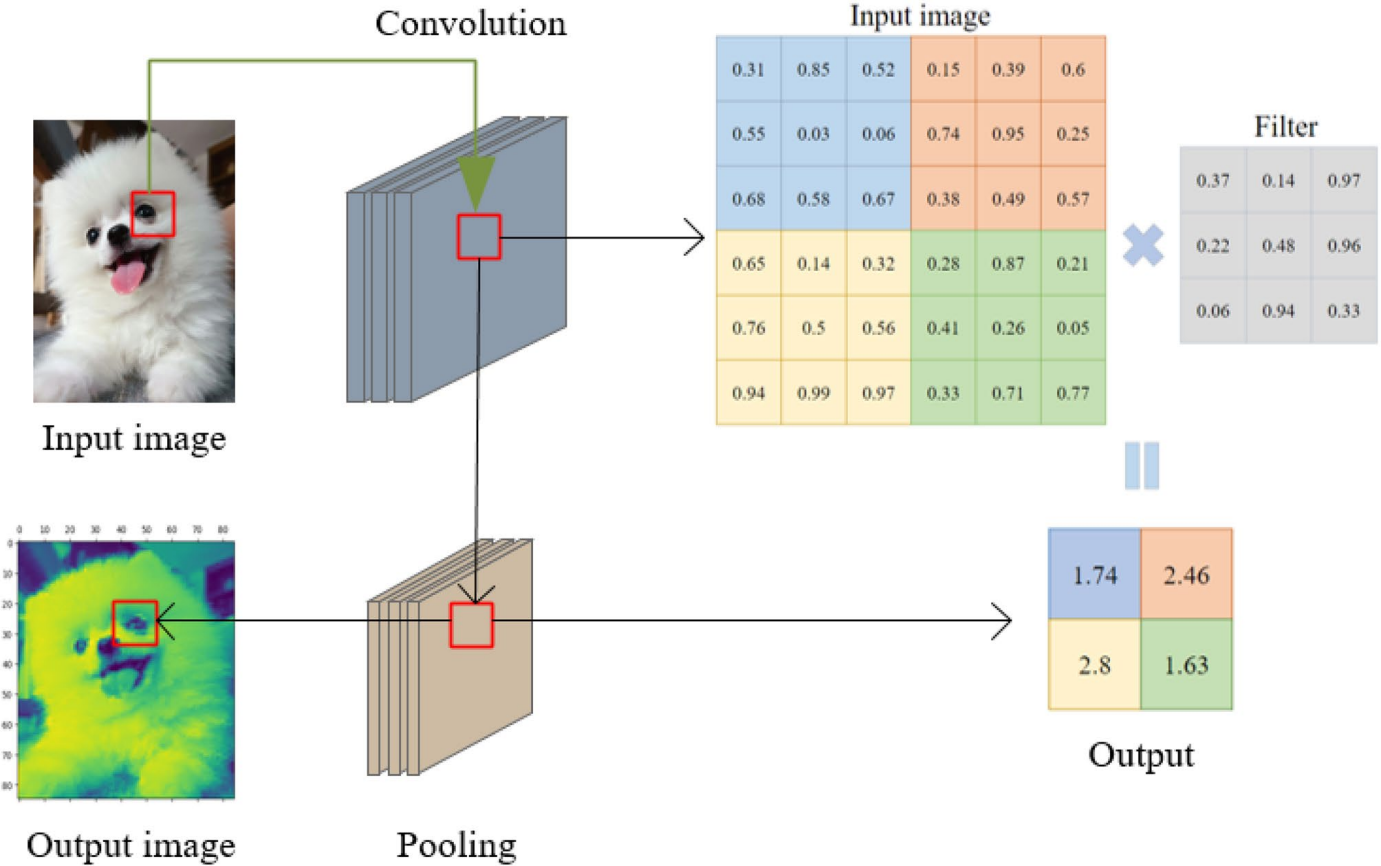
Convolution layer and pooling layer.

The convolutional layer is a key component in the original structure of Convolutional neural network (CNN). It is used for extracting data features, including images, audio^13^, text^14^, time series^15^, and more. By applying filters and creating feature maps, the convolutional layer is able to highlight different features present in the data. These feature maps provide more meaningful representations for the next layer and alter the appearance and shape of the image. The working principle of the convolutional layer is illustrated in Figure 1.

The pooling layer is another important component of convolutional neural networks (CNNs). It is typically used after the convolutional layer to reduce the spatial dimensions of feature maps and decrease the data volume and parameter count. The pooling layer operates by partitioning each feature map into fixed-size regions and performing operations such as selecting the maximum value (max pooling) or calculating the average value (average pooling) within each region.

There are two main types of pooling operations: max pooling and average pooling. Max pooling selects the maximum value within each region as the output, while average pooling calculates the average value. These operations help preserve key features and exhibit a certain degree of translation invariance, making the network more robust to small variations in the input.The pooling layer serves two primary purposes in CNNs. Firstly, it reduces the spatial dimensions of feature maps, reducing computational complexity and shortening training time. Secondly, it extracts essential features and reduces redundant information, thereby improving the model’s generalization ability and interpretability.

The max pooling operation selects the maximum value within each region as the output, thereby ignoring the information of other values. This may lead to the neglect or loss of some critical features, especially in cases involving small-sized features. This information loss can affect the model’s perception of details. Max pooling preserves the maximum value of the input, but it loses the positional information of that maximum value in the feature map. This means that max pooling has certain limitations in preserving positional invariance. In certain applications, positional information may be crucial for accurate classification or segmentation tasks. Max pooling requires selecting an appropriate region size for the operation. Choosing a larger pooling region can reduce the dimensionality of the feature map and improve computational efficiency but may result in more information loss. Choosing a smaller pooling region can better preserve detailed information but increases computational complexity.

Average pooling takes the average value of pixels within each pooling region, resulting in the loss of fine details in the image. Since multiple pixels are combined into one, the pooled feature map cannot accurately preserve subtle variations and edge information present in the original image. Moreover, average pooling is a spatially invariant operation, meaning that regardless of the position of the feature within the image, the average pooling result will be the same. This may not be desirable in certain cases where positional information is important, such as object detection tasks. Additionally, pooling operations reduce the resolution of the feature map, leading to image blurring. This blurring effect can become more pronounced when using larger pooling regions or stacking multiple pooling layers, ultimately impacting the performance of the model.

Choosing the appropriate pooling type in a CNN model is a challenging decision. It requires a comprehensive understanding of the dataset and making corresponding choices. Another related issue with the pooling layer is how to appropriately consider the characteristics of grouped images during the pooling process.

Selecting the appropriate pooling type is a challenging decision in CNN models. It requires a comprehensive understanding of the dataset and making corresponding choices. Another issue related to pooling layers is how to appropriately consider the characteristics of grouped images during the pooling process. Keeping the loss at a minimum is a key factor in ensuring the success of the model. Therefore, when designing CNN models, it is important to carefully evaluate the characteristics of different pooling methods and make selections based on task requirements and data set features to enhance model performance.Different studies have been conducted in the literature to over­come the limitations of pooling functions used in CNNs. Different studies have been conducted in the literature, including a detailed exploration of the limitations associated with using pooling functions. These studies are extensively explained in the literature review section. Hyun et al.^16^ introduced a novel pooling method called Universal Pooling (UP). UP performs different pooling functions based on the training samples and is inspired by attention mechanisms. This method can be regarded as a channel-wise local spatial attention module and is distinct from attention-based feature reduction methods. UP has the capability to train complex pooling functions and outperforms previous pooling methods in terms of performance. However, it should be noted that UP entails a significant computational cost. Williams et al.^17^ proposed a novel approach called Wavelet Pooling, which introduces wavelet pooling as an alternative to traditional neighborhood pooling in the field of convolutional neural networks. This method involves decomposing the input features into a second-level decomposition and discarding the first-level subbands to reduce the feature dimensionality. By doing so, Wavelet Pooling tackles the issue of overfitting often encountered with max pooling while compacting the features in a more efficient manner compared to neighborhood pooling. Experimental results on standard classification datasets demonstrate its impressive performance. However, it should be noted that Wavelet Pooling involves substantial computational complexity, which may pose difficulties in its practical implementation.

In general, pooling methods proposed in similar studies often suffer from issues such as complexity, lack of flexibility, slow speed, and difficulty of use. Moreover, they may have some drawbacks, including slowing down the training and prediction processes of the model instead of accelerating them. This research aims to provide a new and concise approach to minimize information loss caused by pooling layers.

Inspired by the Avg-TopK^18^ method proposed by Cüneyt et al. we incorporate a parameter T to capture the significant features within the pixels with the highest representation and further enhance accuracy. This approach comprehensively addresses the limitations of both max pooling and average pooling.

LeNet-5^19^ is a classic convolutional neural network model proposed by Yann LeCun^19^. As shown in Fig. 8, It consists of five different layers, including two convolutional layers, two pooling layers, and one fully connected layer. It was designed for handwritten digit recognition tasks and has been widely used in image classification, edge detection, and object recognition, among other fields. The advantages of LeNet-5 lie in its simple and effective architectural design and parameter sharing mechanism. Through multiple layers of convolution and pooling operations, LeNet-5 can extract local features from images and reduce the amount of data, thereby speeding up training and improving classification performance. This laid the foundation for the development of more complex convolutional neural network models in the future.

Proposed a new pooling method aimed at overcoming the limitations of traditional pooling functions. This new method addresses the problems commonly seen in CNN networks with max pooling and average pooling layers. Our primary objective is to prevent the loss of highly representative values that may be disregarded by traditional pooling methods and ensure these values are appropriately represented. To achieve this, we introduce K pixels with the highest representational capacity and incorporate a learning parameter T to compute the maximum and average values of these pixels based on crucial feature information. Our strategy aims to alleviate the drawbacks of max pooling and average pooling methods while eliminating noise. We conducted experiments on three different benchmark datasets (CIFAR-10, CIFAR-100, and MNIST) to compare this new pooling method with traditional pooling methods.

The proposed pooling method is expected to have a positive impact on the expansion and development of the convolutional neural network field, especially in tasks that require accurate preservation of features. Additionally, by offering an alternative to the drawbacks of traditional pooling methods, the proposed pooling approach aims to contribute to the improvement and advancement of existing techniques in the field. Contributions of the study:

- A study on the effects of T-Max-Avg, Avg-TopK, and Max-Avg pooling models on grayscale and color images we conducted. We observed that accurately summarizing these features plays a significant role in improving the performance of the model, especially in tasks such as classification.
- Like traditional pooling methods, the T-Max-Avg method is simple, user-friendly, and exhibits good robustness. It offers advantages in terms of cost and speed, making it a favorable choice.
- The T-Max-Avg method does not impose additional overhead on CNN models. It does not increase computational load while enhancing the robustness of the model.
- According to extensive experimental research using different datasets, the T-Max-Avg method has shown higher accuracy compared to Avg-TopK, maximum, and average pooling methods. This indicates that the T-Max-Avg method can more accurately capture feature information and provide better results during the model training process.
- The proposed pooling method aims to address the shortcomings of traditional pooling methods and provide an alternative choice for the development and expansion of existing methods in the field.

The remaining structure of this paper is as follows: Chapter 2 provides a brief review of previous work on pooling layers. Chapter 3 discusses the proposed method in detail. Chapter 4 introduces the experimental study and results. Chapter 5 presents subsequent expansion experiments. Finally, the paper concludes with a discussion and conclusion.

## Literature review

The following are the pooling methods proposed by researchers as alternatives to traditional algorithms in the field of pooling layers. Bilinear pooling, proposed by Lin et al.^20^, is a commonly used pooling method in deep learning. It extracts the second-order relationships between features by computing the outer product of two input feature maps. This method is able to better capture the spatial relative positions and interactions between features.

Lee et al.^21^ proposed three new pooling methods: hybrid pooling, gate pooling, and tree pooling. Hybrid pooling combines different pooling functions and dynamically selects which pooling operation to apply to each pixel based on the specific requirements. Gate pooling uses gating mechanisms to dynamically determine which pooling function to apply to each pixel. Tree pooling uses a tree structure to partition feature maps and capture hierarchical feature information. These new pooling methods offer more flexible and diverse ways of pooling, enabling more effective extraction of important information from images or features.

Detail preserving pooling^22^, proposed detail preserving pooling (DPP), an adaptive pooling method that preserves important^23^ structural details. This method utilizes the concept of inverse binary filters. DPP enables downsizing to focus on critical structural details; learnable parameters control the amount of detail protection.

Stergiou et al.^24^ proposed an adaptive exponential weighted pooling method called adaPool. This method learns a region-specific fusion of two sets of pooling kernels based on the Dice–Sørensen coefficient and the exponential maximum. adaPool improves the preservation of details in various tasks such as image and video classification, as well as object detection. One key feature of adaPool is its bidirectional nature, where the learned weights can also be utilized for upsampling activation maps. adaPool consistently achieves good experimental results across tasks and backbone structures. Zhang et al.^25^ proposed a novel end-to-end trainable global pooling operator called AlphaMEX Global Pool, which utilizes a nonlinear smooth logarithmic mean exponential function, AlphaMEX, to effectively extract features and enhance network intelligence. Compared to the original global pooling layer, our proposed method improves classification accuracy without the need for additional layers or excessive redundant parameters. Experimental results on CIFAR-10/CIFAR-100, SVHN, and ImageNet demonstrate the effectiveness of this approach.

Lee et al.^26^ proposed a graph pooling method based on self-attention. By combining graph convolution with self-attention, their method can simultaneously consider node features and graph topology. To ensure a fair comparison, they conducted experiments using the same training procedure and model architecture for existing pooling methods and their proposed method. The experimental results demonstrate that their method achieves superior graph classification performance on benchmark datasets with a reasonable number of parameters.

One of the latest pooling methods is the vector pooling block (VPB)^27^, proposed by Mohamed et al. The VPB consists of two data paths, primarily extracting features in the horizontal and vertical directions. Unlike traditional pooling layers that use fixed square kernels, the VPB employs longer and narrower pooling kernels, enabling the convolutional neural network (CNN) to gather both global and local features simultaneously. The new Avg-TopK pooling model proposed by Cüneyt et al.^18^ selects the top K pixels with the highest interactions and averages them. This model is designed to address the limitations of max pooling and average pooling.

## Method

CNN is widely used in computer vision applications. Its main function in these applications is to automatically extract image features. In the convolutional layers of CNN, the input values undergo filtering operations to extract the features of the image. Through these filtering operations, CNN can identify unique features within the image. The output of the convolutional operation is referred to as feature maps. Fig. 1 illustrates the process of applying convolutional operation to a 6 × 6 image data. After the convolutional operation, the output is passed to the pooling layer for further processing.

### Pooling layer

In the CNN architecture, there is no direct connection allowing information to flow from the next layer back to the previous layer. This implies that there is no direct communication between the layers. However, for the success of the model, it is crucial to transmit valuable information to the next layer. Our experimental results will compare the average, maximum, and Avg-TopK pooling methods.

#### Average pooling

Average pooling^28^ is a commonly used feature extraction operation that is widely applied in convolutional neural networks. It reduces the spatial dimensions of features by calculating the average value of each small window or region. Specifically, for each small window or region, average pooling computes the average value of all the values within it and replaces the original data with this average value. However, due to the adoption of the average operation, it may not effectively handle subtle feature variations or significant features present in certain image regions. The formula for this method is shown as Eq. (1).

1 $$\begin{aligned} \textrm{F}_{\text{ average } (\textrm{x})}=\frac{1}{N} \sum _{i=1}^{N} x_{i} \end{aligned}$$

where x is the values of the input image in the pooling region.

**Figure 2:**
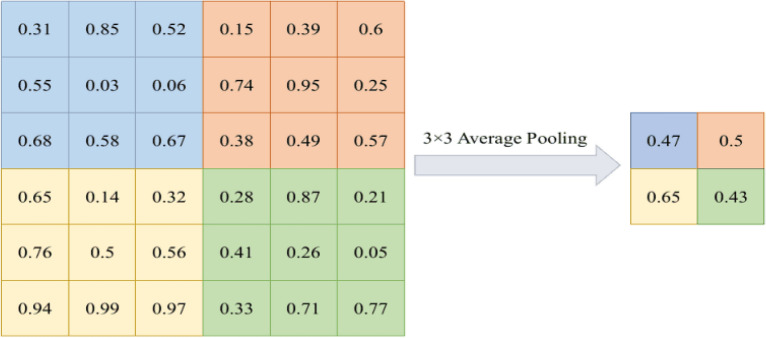
3$$\times$$3 Average pooling.

Figure 2 shows the 2 $\times$ 2 matrix produced by applying average pooling to a 6 × 6 pixel input with a 3 $\times$ 3 filter size.

#### Max pooling

Max pooling is a commonly used feature extraction operation, typically applied in convolutional neural networks. It reduces the spatial dimensions of features by selecting the maximum value within each small window or region. Specifically, for each small window or region, max pooling selects the maximum value and replaces the original data with it. This operation helps to preserve the most prominent features in an image, such as edges and textures. Compared to average pooling, max pooling is better at capturing local feature information. As a result, max pooling^29^ is widely used in image processing and computer vision tasks.

In the process of max pooling, only the maximum value within a small window or region is selected as a representative, while discarding other detailed information. This operation leads to partial information loss in the original data and reduces the accuracy of features. The mathematical expression of this method is shown as Eq. (2).

2 $$\begin{aligned} \textrm{F}_{\max (\textrm{x})}=\max \left\{ x_{i}\right\} _{i=0}^{N} \end{aligned}$$

where x is the values of the input image in the pooling region.

**Figure 3:**
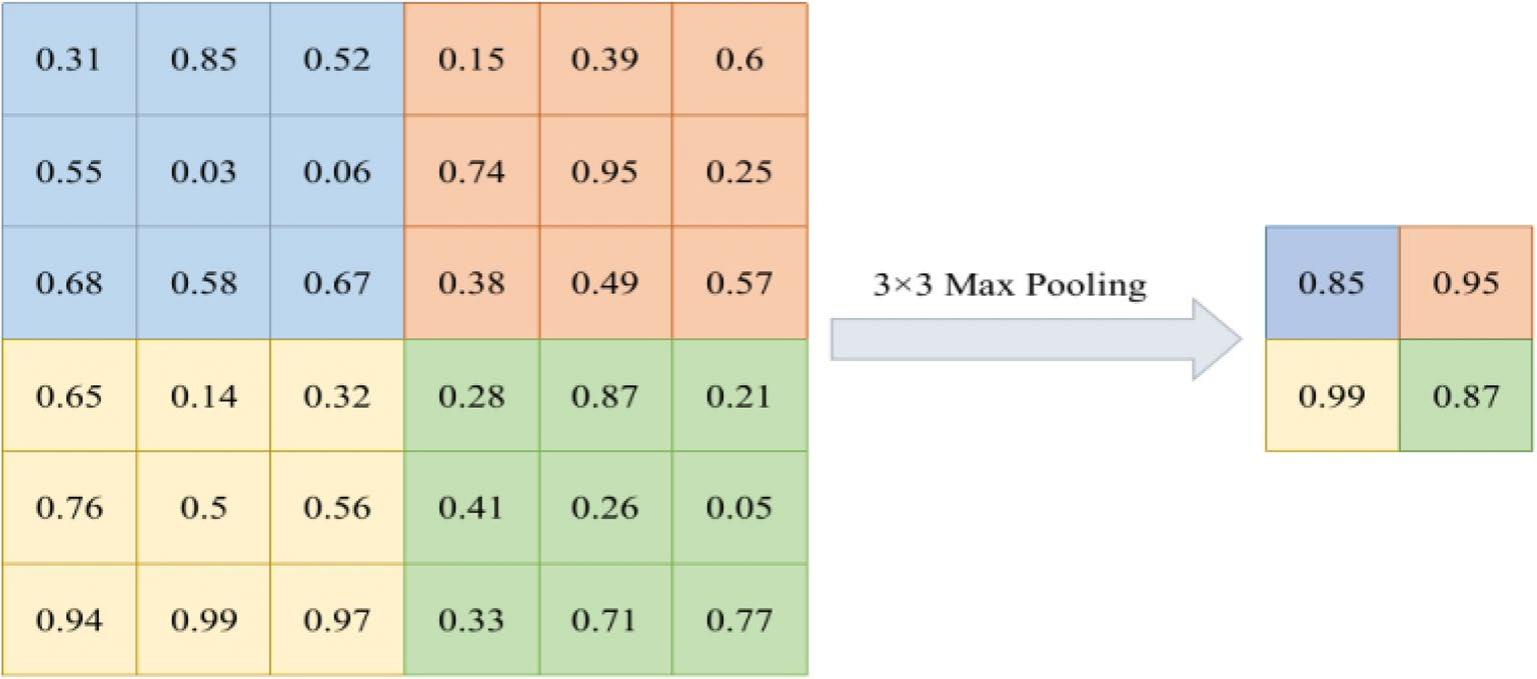
3$$\times$$3 Max pooling.

The 2 $\times$ 2 matrix obtained when a maximum pooling of 3 $\times$ 3 filter size is applied on a 6x6 pixel is shown in Figure 3.

#### Avg-TopK pooling

The Avg-TopK method aims to eliminate the drawbacks of both max pooling and average pooling methods. It is an approximate value that represents all pixels by selecting the top k highest entries from the input data. The average value of the k pixels with the highest values is determined. Equation. (3) represents the mathematical expression of the Avg-TopK pooling method.

3 $$\begin{aligned} \textrm{F}_{\textrm{Avg}-\textrm{TopK}(\textrm{X})}=\frac{1}{\textrm{k}} \sum _{\textrm{i}=1}^{\textrm{k}} \textrm{Y}_{\textrm{i}} \end{aligned}$$

X represents the set of elements with pixels according to the pool size value selected from the data coming from the convolution layer. Yi represents the i-th highest pixel value. F(Avg-TopK) represents the average of the k highest items.

**Figure 4:**
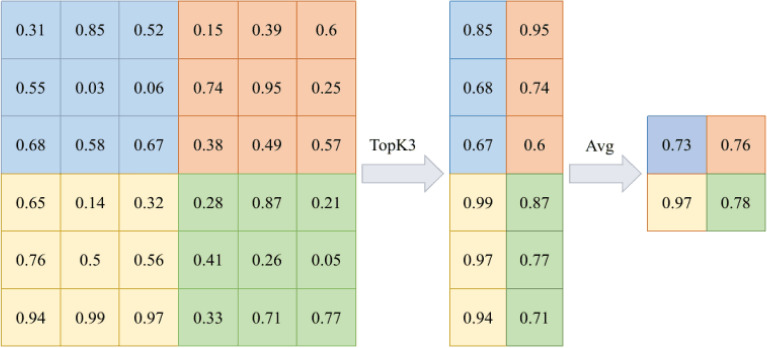
3$\times$3 Avg-TopK pooling.

Figure 4 shows the operations for K = 3 in Avg-TopK pooling operation with a filter size of 3 × 3 from 6 × 6 pixel input.

#### Methodology suggested for pooling

Both the Avg-TopK method and the Avg pooling method aim to address the drawbacks of the Max pooling and Average pooling methods. In the Avg pooling method, both highly representative values and minimally representative values are treated equally. This ensures that in the presence of values close to zero, the result is close to zero and dominant values do not receive their deserved values. In Max pooling, the highest representative value is selected while ignoring all other values, which can significantly impact classification performance when the highest value is a noisy pixel point. To address these two issues in our proposed pooling model, we take multiple highly representative high values into account and incorporate a parameter T, with its value range between [0 and 1]. This parameter controls whether the maximum value or the average value of these highly representative values is taken. Thus, it resolves the issue of input close to zero in the Avg pooling and the problem of noisy pixel points in Max pooling. Moreover, this method can adapt to various image datasets through the parameter T, and it can represent an approximate value for all pixels.

In our proposed method, we continuously select the K highest pixel values from the input data. We incorporate a parameter T to control the output of the average and maximum values of these K highest pixel values. The proposed method is called T-Max-Avg. Eq. (4) represents the mathematical expression of the T-Max-Avg pooling method.

4 $$\begin{aligned} \textrm{F}_{\textrm{T}_{\_} {\text {Max}}_{\_} {\text {Avg}}(\textrm{X})}=\left\{ \begin{array}{ll}\max \left\{ \textrm{Y}_{\textrm{i}}\right\} _{\textrm{i}=0}^{\textrm{K}} &{} ,\quad \textrm{Y}_{\textrm{i}} \ge \textrm{T} \\ \frac{1}{\mathrm {~K}} \sum _{\textrm{i}=1}^{\textrm{K}} \textrm{Y}_{\textrm{i}} &{} ,\quad \textrm{Y}_{\textrm{i}}<\textrm{T}\end{array}\right. \end{aligned}$$

X represents the set of elements with pixels selected based on the pool size values obtained from the data from the convolutional layer. Yi represents the i-th largest item, T represents the parameter ranging from 0 to 1, K represents the number of high-value items, and F(T-Max-Avg(X)) represents the final result.

**Figure 5:**
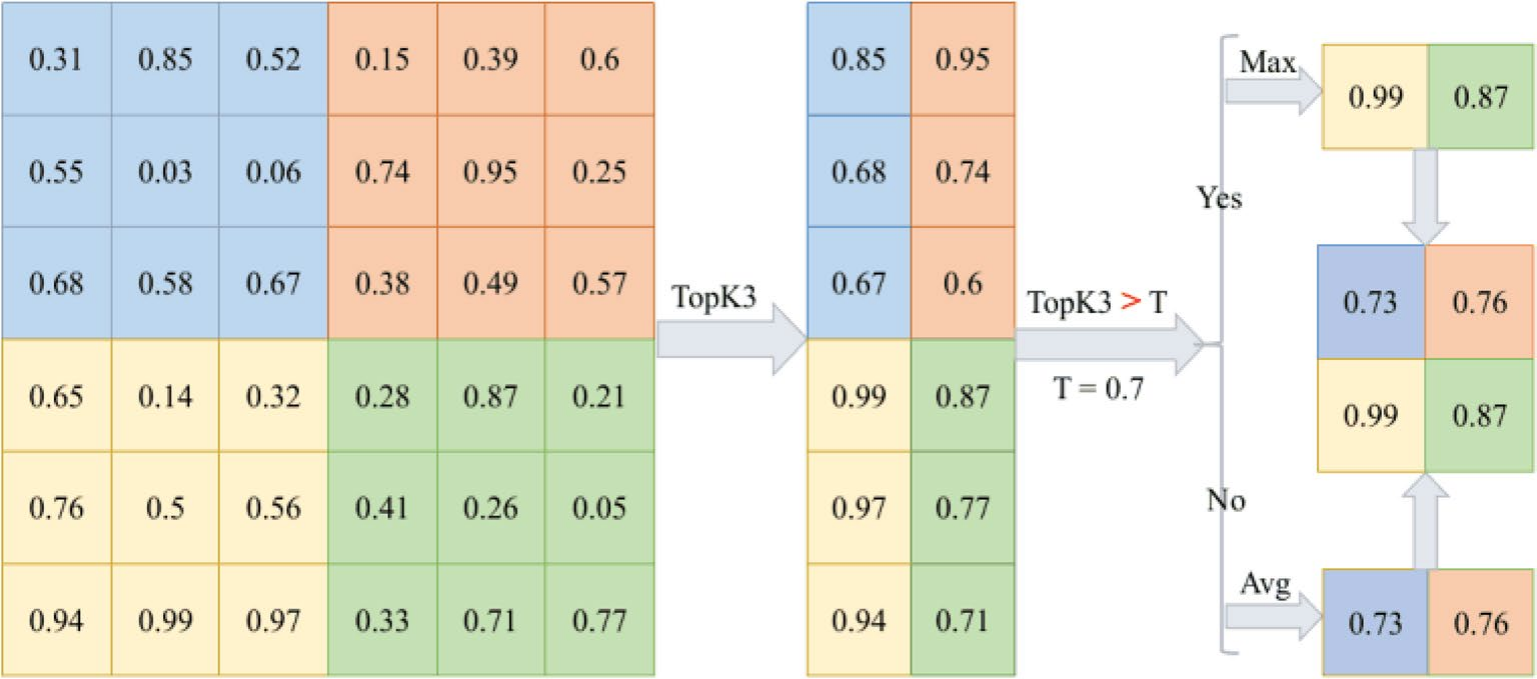
3$\times$3 T-Max-Avg pooling.

Figure 5 shows the operations for K = 3 in T-Max-Avg pooling operation with a filter size of 3x3 from 6x6 pixel input.

**Figure 6:**
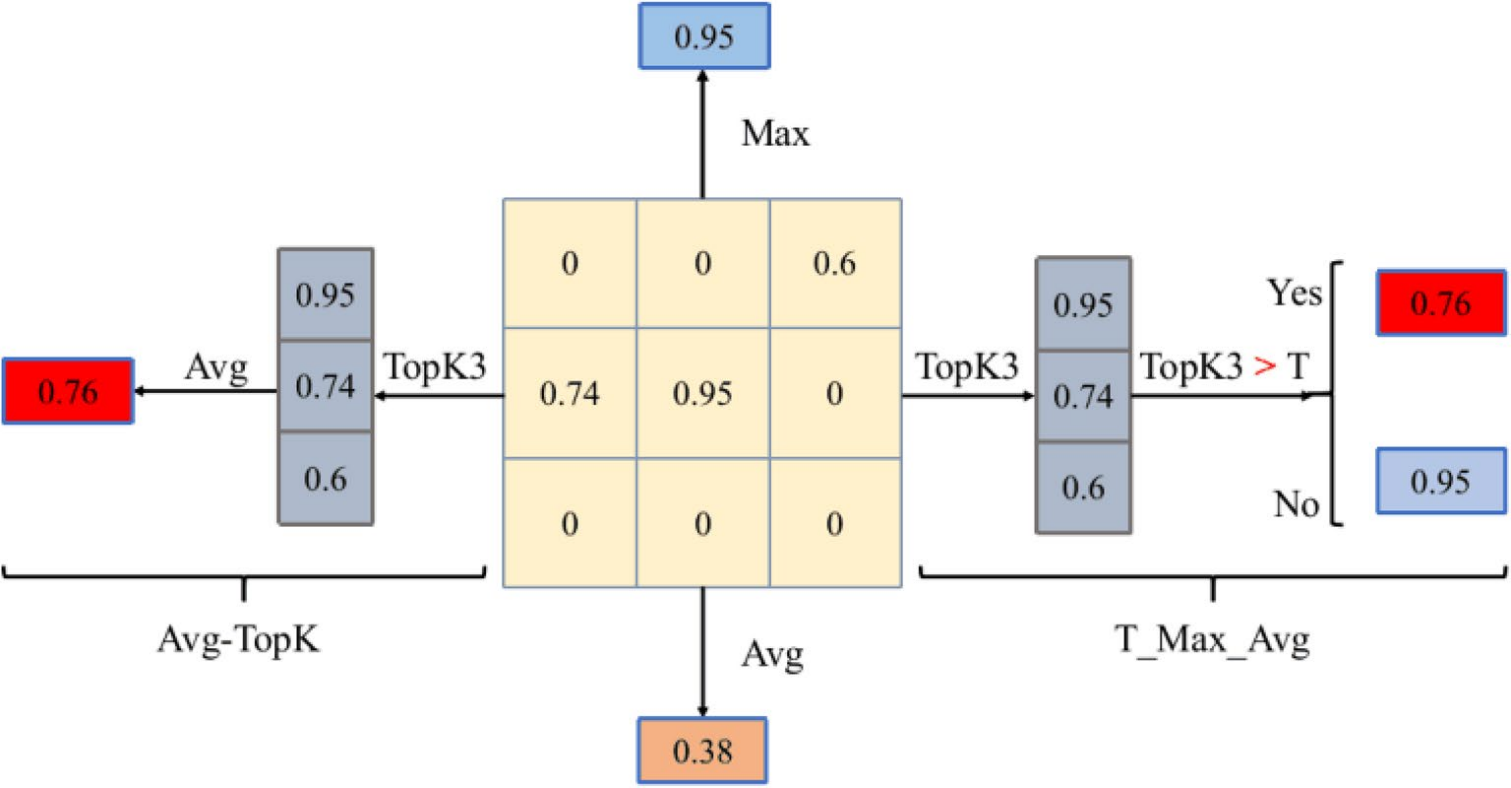
T-Max-Avg, Avg-TopK, Max, Average with 3$\times$3 pooling method.

In Figure 6, the results of the average, max, Avg-TopK(K=3) and T-Max-Avg(K = 3) pooling method applied to a 3 $\times$ 3 data are shown visually.

These examples demonstrate that the recommended T-Max-Avg method provides values based on both maximum pooling and Avg-TopK. The values obtained from this method can extract the most important information from the image to a great extent.

**Figure 7:**
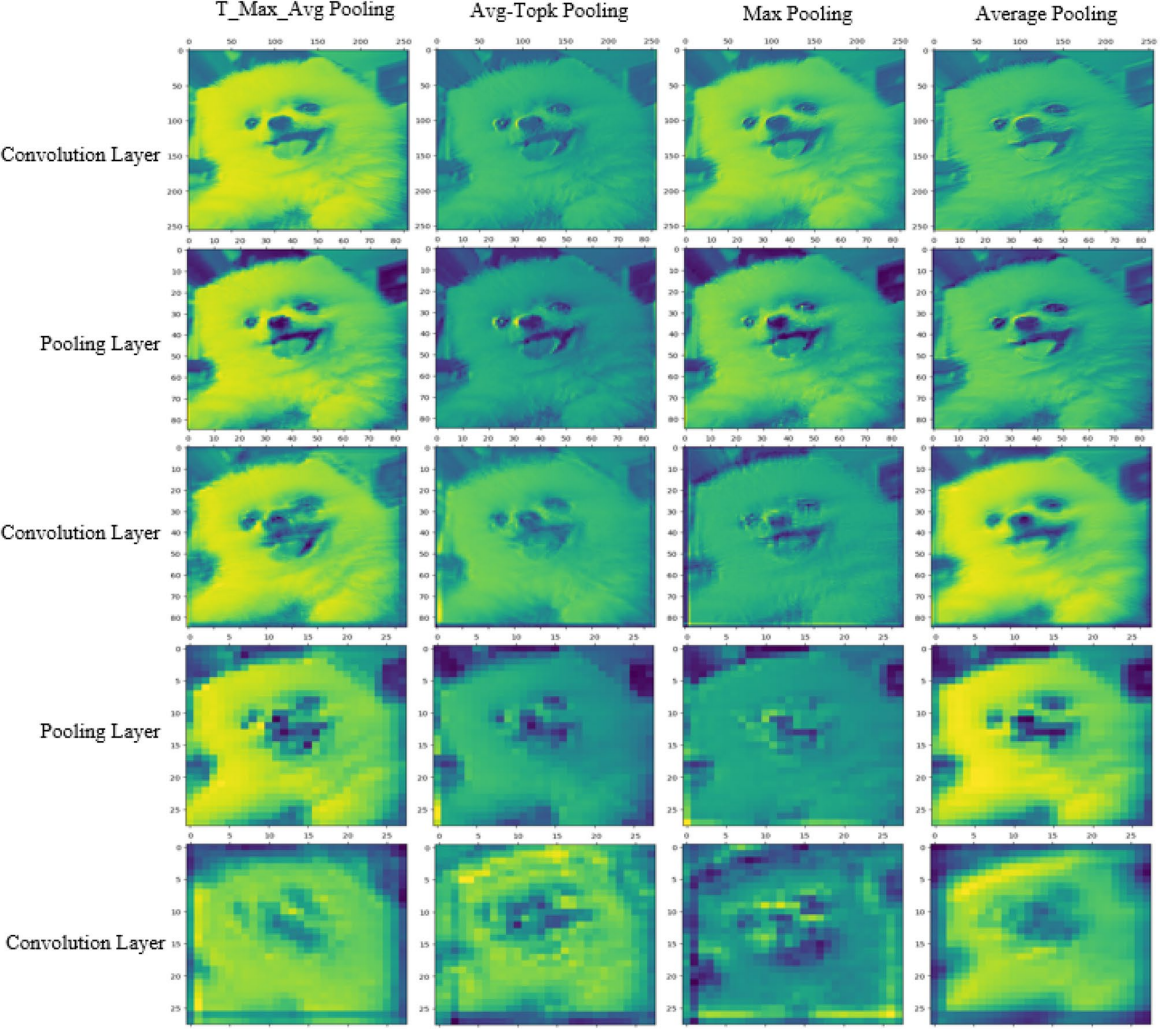
Visualization of T-Max-Avg, Avg-TopK, Max and Average Pooling method on a sample image.

Figure 7 shows the image transformation based on the selected pooling layer when applying 3 convolutions (1 filter) and 2 pooling layers (LeNet5) to an example image. The pool size is considered to be 3x3. From the figure, it can be observed that the shape of the image changes corresponding to the pixel values based on different pooling methods.

### Created CNN network

**Figure 8:**
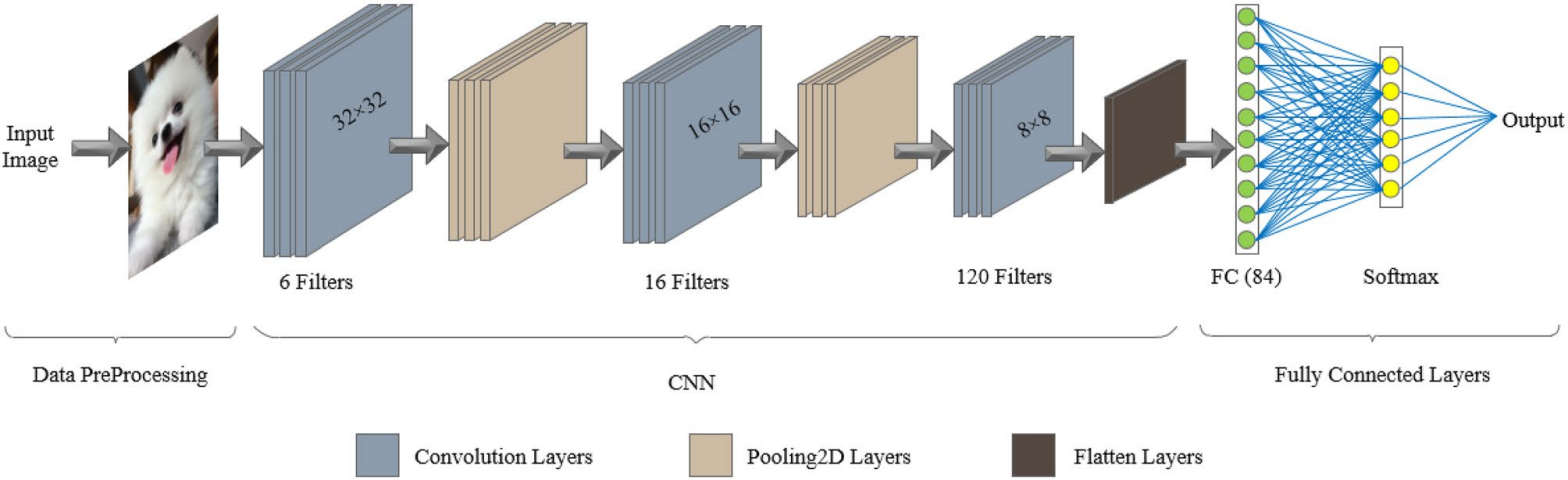
LeNet-5 model architecture.

To evaluate the effectiveness of our proposed pooling layer, we conducted experiments using the same model, dataset, and parameters as the Avg-TopK method. Therefore, we chose the LeNet-5^19^ convolutional neural network and a public dataset. LeNet-5 was selected as the preferred option due to its simple structure and powerful classification capabilities. It is a traditional CNN architecture that has significantly contributed to the development of CNNs. The network model was initially proposed by LeCun et al.^19^. The LeNet-5 network structure consists of a total of 7 layers, including 3 convolutional layers, 2 pooling layers, and 2 fully connected layers. Table 1 shows the architecture of the LeNet-5 convolutional neural network. Figure 8 illustrates the CNN architecture used in the experimental study.

## Experimental results

### Dataset

In this section, we will explain the datasets used to compare the performance of the proposed model with traditional methods. These datasets include publicly available MNIST, CIFAR-10, and CIFAR-100 datasets.

#### CIFAR-10 dataset

The CIFAR-10 dataset is a classic dataset widely used for computer vision tasks. It contains 60,000 color images divided into 10 different classes, with each class having 6000 images. The classes include airplanes, cars, birds, cats, deer, dogs, frogs, horses, ships, and trucks. Each image has a size of 32 × 32 pixels and consists of RGB channels. This dataset is commonly used to evaluate the performance of models in tasks such as image classification, object detection, and image generation in the field of computer vision. Due to its relatively small scale and diverse categories, CIFAR-10 has become one of the benchmark datasets extensively used in both academia and industry.

#### CIFAR-100 dataset

The CIFAR-100 dataset is a classic dataset widely used for computer vision tasks. It is an extended version of the CIFAR-10 dataset, consisting of 100 different fine-grained categories. Each category has 600 images, resulting in a total of 60,000 images. These categories cover a wide range of objects, animals, and everyday items such as chairs, tables, flowers, insects, and more. Similar to CIFAR-10, each image in CIFAR-100 has a size of 32 × 32 pixels and consists of RGB channels.

#### MNIST dataset

The MNIST dataset^30^ is a classic handwritten digit recognition dataset widely used in machine learning and computer vision research and education. It consists of a collection of samples representing digits from 0 to 9, with each sample being a grayscale image of size 28x28 pixels.

The MNIST dataset contains 60,000 training samples and 10,000 testing samples. The training samples are used to train models, while the testing samples are used to evaluate model performance. These samples are handwritten by different individuals, covering various styles and variations. Therefore, the MNIST dataset is valuable for assessing the robustness and generalization ability of models in digit recognition tasks.

### Network parameters

In this section of experimental analysis, all experiments were conducted with the same settings as Avg-TopK. The “man” algorithm and its default parameters were used in the gradient-based optimization algorithm. The pixel values of the images were scaled from 0–255 to 0–1 to normalize the dataset. The model was trained for a total of 50 epochs. Additionally, the stride value and parameters of all other network layers were set to their default values.

### Experimental results

Three different datasets and the LeNet-5 CNN model were used for experimental research. The model settings are as described above. To evaluate the performance of the model on grayscale and color images, different datasets were used. In the CNN architecture, pooling layers are commonly used with a scale of 2 and 3. In this study, additional experiments were conducted using a pooling scale of 4, but it was found to have little significance beyond that.

**Table 1:** LeNet-5 network architecture.

Layer	Layer type	Size of feature map	Kernel size	Param	Activation functions
Input layer	Image	32$\times$32$\times$3	–	–	–
Conv1 layer	Convolution	32$\times$32$\times$6	5$\times$5	456	Tanh
Pool1 layer	CustomPooling	16$\times$16$\times$6	2$\times$2	0	–
Conv2 layer	Convolution	16$\times$16$\times$16	5$\times$5	2416	Tanh
Pool2 layer	CustomPooling	8$\times$8$\times$16	2$\times$2	0	–
Conv3 layer	Convolution	8$\times$8$\times$120	5$\times$5	48,120	Tanh
FC1 layer	Full connected	84	–	645,204	Tanh
FC2 lyer	Full connected	10	–	850	Softmax

During the experiments with the LeNet-5 model, we used Google Colab, and for the extended experiments, we utilized a device with NVIDIA GeForce GTX 1050. Since the method in this paper selects the same model and parameters as the Avg-TopK pooling method for training, the experimental results obtained using Avg-TopK, max pooling, and average pooling methods are consistent, and there is no need to repeat the experiments. The experimental results were averaged over 6 runs. Following the experience from Cüneyt et al.^18^, this paper adopts a Pool Size of 4 and K equal to 6 on all three datasets to fine-tune the learning parameter T. The fine-tuning results are shown in Figure 12.

In studies conducted with pooling size 2, the accuracy values of the traditional and proposed pooling method on the data sets are shown in Tables 2,3 and 4.

**Figure 9:**
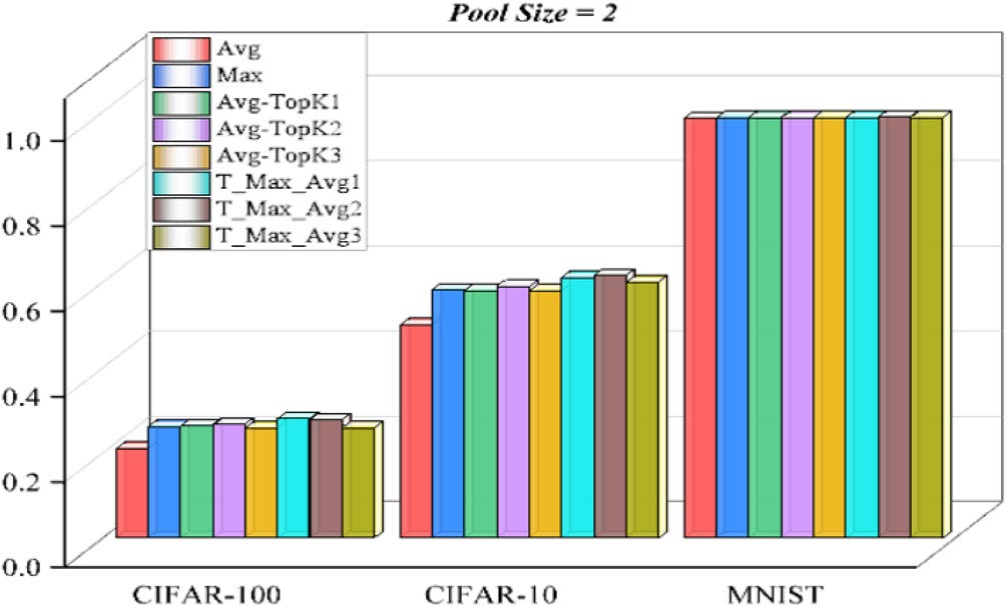
Success of pooling methods on datasets for Pooling size 2.

The visual expression of the results obtained in Tables 2,3 and 4 is given in Figure 9.

As can be seen from the experimental results with Pool size 2, T-Max-Avg method:

- According to observations, the max pooling method has shown significantly more success than the average pooling method on three different datasets.
- The general average value (K = 2) of T-Max-Avg method, as described in the experiments, has shown more success than Avg-TopK and traditional pooling methods. Considering the highest results in the experimental results with Pool size 2, Avg-Top2 method:
- It has been determined that the method in this paper improves the average accuracy on CIFAR-10 by 2.45% compared to the Avg-TopK pooling method, by 3.44% compared to the max pooling method, and by 11.45% compared to the average pooling method.
- It has been determined that the method in this paper improves the average accuracy on CIFAR-100 by 1.73% compared to the Avg-TopK pooling method, by 1.93% compared to the max pooling method, and by 6.93% compared to the average pooling method.
- We have confirmed that the T-Max-Avg method improves the overall average accuracy of the MNIST dataset by 0.2% compared to the Avg-TopK pooling method. It achieves an average increase of 0.12% in overall accuracy compared to the max pooling method, and a 0.84% improvement compared to the average pooling method.

**Table 2:** Experimental results with Pool-size = 2 and T = 0.7 on CIFAR-100 dataset.

CIFAR-100	Exp1 (%)	Exp2 (%)	Exp3 (%)	Exp4 (%)	Exp5 (%)	Exp6 (%)	Average (%)	Avg-TopK(Average) (%)
Avg	21.9	21.5	25	20.2	21.4	21.14	21	–
Max	26.6	25.7	26.1	26.2	25.5	26.46	26.1	–
T-Max-Avg1	28.01	27.77	28.66	27.39	28.39	27.97	28.03	26.3
T-Max-Avg2	28.01	27.74	28.28	27.64	27.67	26.65	27.67	26.63
T-Max-Avg3	26	26.14	25.37	26.49	26.07	25.29	25.89	25.8

**Table 3:** Experimental results with Pool-size = 2 and T = 0.7 on CIFAR-10 dataset.

CIFAR-10	Exper1 (%)	Exp2 (%)	Expt3 (%)	Exp4 (%)	Exp5 (%)	Exp6 (%)	Average (%)	Avg-TopK(Average) (%)
Avg	49	49.9	49	50.2	50.69	50.16	50	-
Max	57	56.7	57.3	57	60.37	60.02	58.1	-
T-Max-Avg1	60.55	60.97	60.87	61.78	60.36	61.1	60.94	58
T-Max-Avg2	61.68	60.58	61.38	61.84	61.85	61.17	61.45	59
T-Max-Avg3	59.9	60.19	60.42	59.59	60.14	58.9	59.86	58

**Table 4:** Experimental results with Pool-size = 2 and T = 0.8 on MNIST dataset.

MNIST	Exp1 (%)	Exp2 (%)	Exp3 (%)	Exp4 (%)	Exp5 (%)	Exp6 (%)	Average (%)	Avg-TopK(Average) (%)
Avg	98.38	98.52	98.3	97.96	98.36	98.75	98.38	–
Max	98.63	98.71	98.51	98.59	98.61	98.67	98.62	-
T-Max-Avg1	98.62	98.36	98.68	98.6	98.58	98.71	98.6	98.58
T-Max-Avg2	98.89	98.66	98.61	98.75	98.71	98.78	98.73	98.58
T-Max-Avg3	98.77	98.58	98.57	98.73	98.7	98.52	98.65	98.63

In the experimental study, when the pooling size is 3, we obtained the experimental results of the Avg-TopK, Max, Average, and the proposed pooling models.

**Figure 10:**
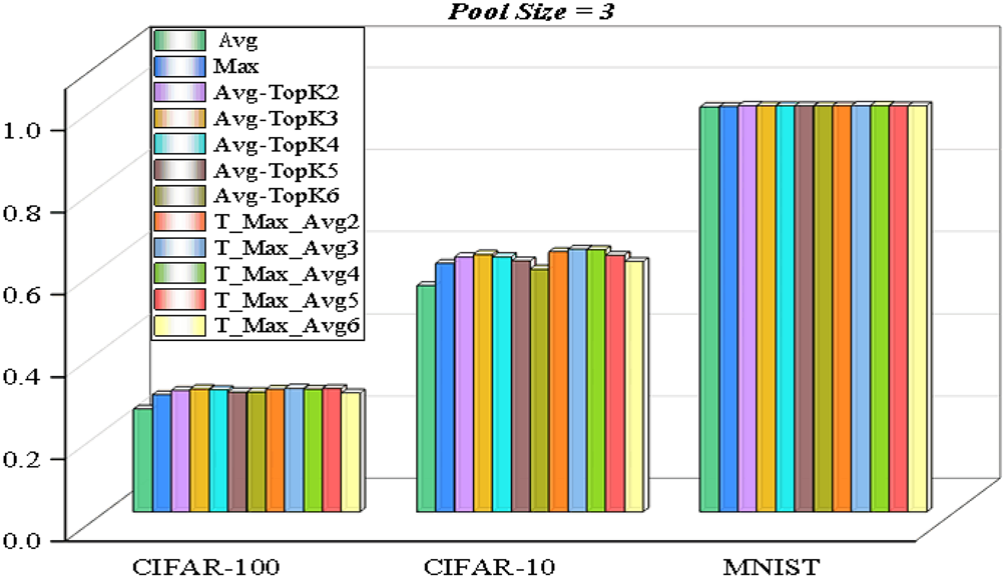
Success of pooling methods on datasets for Pooling size 3.

The visual expression of the results obtained in Tables 5, 6 and 7 is given in Figure 10.

In the experimental results with pool size 3:

- The max pooling method performed significantly better than the average pooling method in the CIFAR-10 and CIFAR-100 datasets,while the average pooling method was slightly more successful than the max pooling method in the MNIST dataset. The max pooling method performed significantly better than the average pooling method in the CIFAR-10 and CIFAR-100 datasets, Considering the highest results in the experimental results with Pool size 3, T-Max-Avg method:
- It has been determined that our proposed method achieves better results than the other three methods on the MNIST dataset with a pool size of 3. ·Compared to the Avg-TopK pooling method, the T-Max-Avg method improves the average best accuracy of the CIFAR-10 dataset by 1.23%. It achieves an average increase of 3.43% in the best accuracy compared to the max pooling method, and an improvement of 8.83% compared to the average pooling method.
- Compared to the Avg-TopK pooling method, the T-Max-Avg method decreases the average best accuracy of the CIFAR-100 dataset by 0.3%. It achieves an average increase of 1.6% in the best accuracy compared to the max pooling method and an improvement of 5.1% compared to the average pooling method.
- We have confirmed that the T-Max-Avg method improves the overall average accuracy of the MNIST dataset by 0.24% compared to the Avg-TopK pooling method. It achieves an average increase of 1.05% in overall accuracy compared to the max pooling method, and a 1.35% improvement compared to the average pooling method.

**Figure 11:**
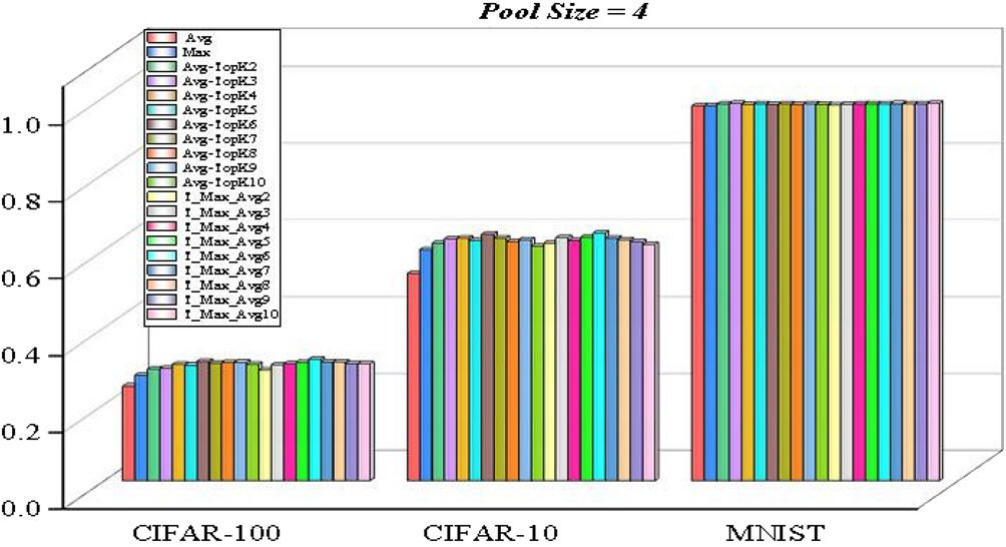
Success of pooling methods on datasets for Pooling size 4.

The accuracy values of the T-Max-Avg method and the other three methods on the dataset, with a pool size of 4, are shown in Tables 8, 9 and 10. The accuracy is illustrated in Figure 11.

- It has been observed that the Max pooling method is more successful than the Avg pooling method in all data sets.
- The research findings suggest that the proposed T-Max-Avg method achieves better results than the Avg-TopK, Max, and Average pooling methods when PoolSize = 4 and K = 6.

**Figure 12:**
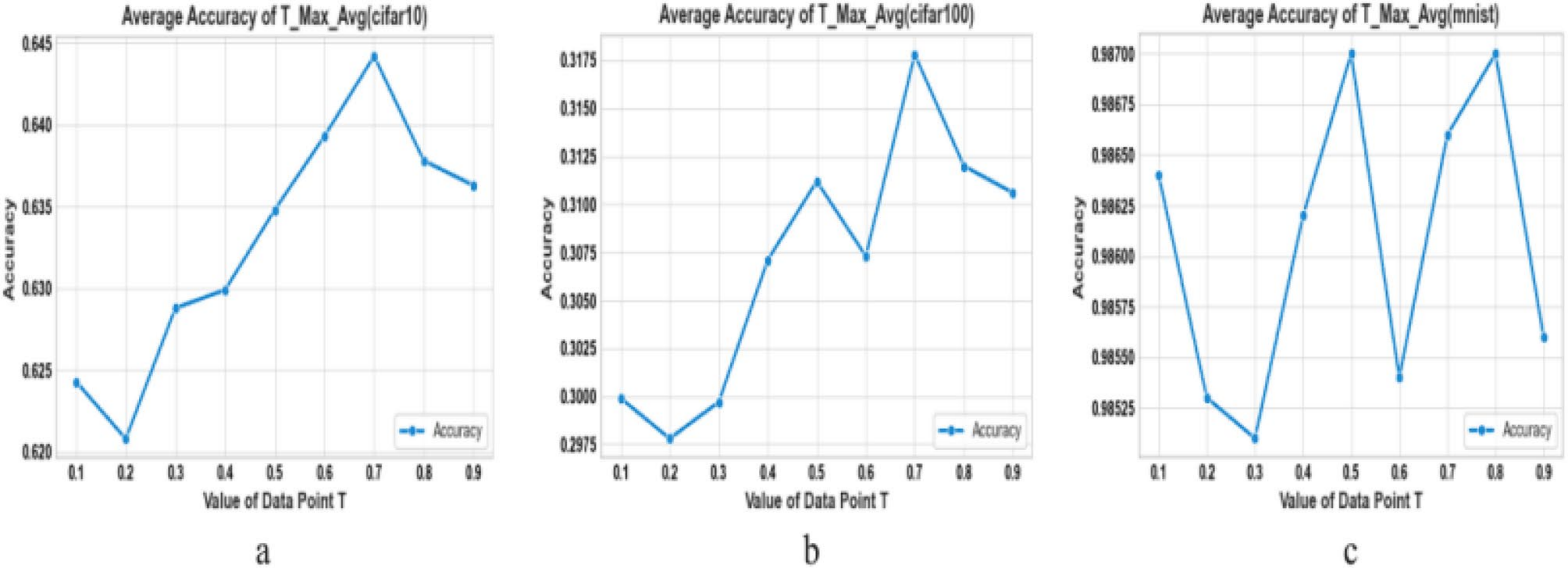
Visualizing parameter T, (**a**) Size = 4 and K = 6, (**b**) Size = 4 and K = 6, (**c**) Size = 2 and K = 2.

Based on experience, we set the pool size to 4 and the K value to 6 for the CIFAR10 and CIFAR100 datasets, and set the pool size to 2 and the K value to 2 for the MNIST dataset to seek the optimal learning parameter T for the T-Max-Avg pooling method. The accuracy results are shown in Tables 11, 12, 13 and Figure 12 provides a visual representation of the optimization process.

**Table 5:** Experimental results with Pool-size = 3 and T = 0.7 on CIFAR-100 dataset.

CIFAR-100	Exp1 (%)	Exp2 (%)	Exp3 (%)	Exp4 (%)	Exp5 (%)	Exp6 (%)	Average (%)	Avg-TopK(Average) (%)
Avg	21.9	21.5	25	20.2	21.4	21.14	25	–
Max	26.6	25.7	26.1	26.2	25.5	26.46	28.5	–
T-Max-Avg2	29.11	30.58	29.98	29.95	29.5	29.9	29.84	29.55
T-Max-Avg3	30.2	30.35	31.17	30.07	29.58	29.25	30.1	29.95
T-Max-Avg4	28.95	29.43	30.08	30.53	29.52	30.71	29.87	29.7
T-Max-Avg5	29.92	30.27	30.11	29.63	30.05	30.44	30.07	29.2
T-Max-Avg6	29.06	28.75	28.79	29.26	29.34	28.39	28.93	29.2

**Table 6:** Experimental results with Pool-size = 3 and T = 0.7 on CIFAR-10 dataset.

CIFAR-10	Exp1 (%)	Exp2 (%)	Exp3 (%)	Exp4 (%)	Exp5 (%)	Exp6 (%)	Average (%)	Avg-TopK(Average) (%)
Avg	55	54.6	54.31	55.7	55	53.84	55	–
Max	61	61.4	59.78	59.5	60.7	60.7	60.4	–
T-Max-Avg2	62.62	64.16	63.01	64.22	63.28	62.18	63.25	62
T-Max-Avg3	64.26	63.66	63.92	63.73	63.83	63.58	63.83	62.6
T-Max-Avg4	64	64.48	63.98	63.06	63.52	63.23	63.71	62
T-Max-Avg5	63.11	62.37	61.71	62.24	62.46	62.54	62.41	61
T-Max-Avg6	61.96	61.84	61	59.24	61.12	59.8	60.83	59

**Table 7:** Experimental results with Pool-size = 3 and T = 0.8 on MNIST dataset.

MNIST	Exp1 (%)	Exp2 (%)	Exp3 (%)	Exp4 (%)	Exp5 (%)	Exp6 (%)	Average (%)	Avg-TopK(Average) (%)
Avg	98.72	98.4	98.36	98.74	98.66	98.52	98.57	–
Max	98.7	98.47	98.46	98.66	98.73	98.74	98.63	–
T-Max-Avg2	98.72	98.87	98.83	98.88	98.77	98.99	98.84	98.87
T-Max-Avg3	98.82	98.93	98.94	98.82	98.81	98.93	98.88	98.81
T-Max-Avg4	98.79	98.85	98.88	98.87	98.93	98.89	98.87	98.76
T-Max-Avg5	98.86	98.89	98.91	98.38	98.81	98.87	98.79	98.78
T-Max-Avg6	98.65	98.73	98.88	98.77	98.93	98.97	98.82	98.74

- We have determined that compared to the Avg-TopK pooling method, the T-Max-Avg method improved the average optimal accuracy on the CIFAR-10 dataset by 0.28%. Compared to the Max pooling method, it improved the average optimal accuracy by 4.32%. Furthermore, compared to the Average pooling method, it improved the accuracy by 10.42
- Based on our findings, the T-Max-Avg method demonstrated a noteworthy enhancement in average optimal accuracy when compared to the Avg-TopK pooling method on the CIFAR-100 dataset, with an improvement of 0.53%. Furthermore, it showcased a significant improvement of 4.11% in average optimal accuracy compared to the Max pooling method. Additionally, there was a remarkable boost in accuracy by 6.96% when compared to the Average pooling method.
- After conducting our analysis, we found that the T-Max-Avg method resulted in an average optimal accuracy improvement of 0.01% compared to the Avg-TopK pooling method on the MNIST dataset. Additionally, it showed an average optimal accuracy improvement of 0.43% when compared to the Max pooling method. Moreover, there was a 0.44% improvement in accuracy compared to the Average pooling method.

Table 14 displays the top accuracy scores achieved from the experimental studies conducted on the datasets.

**Figure 13:**
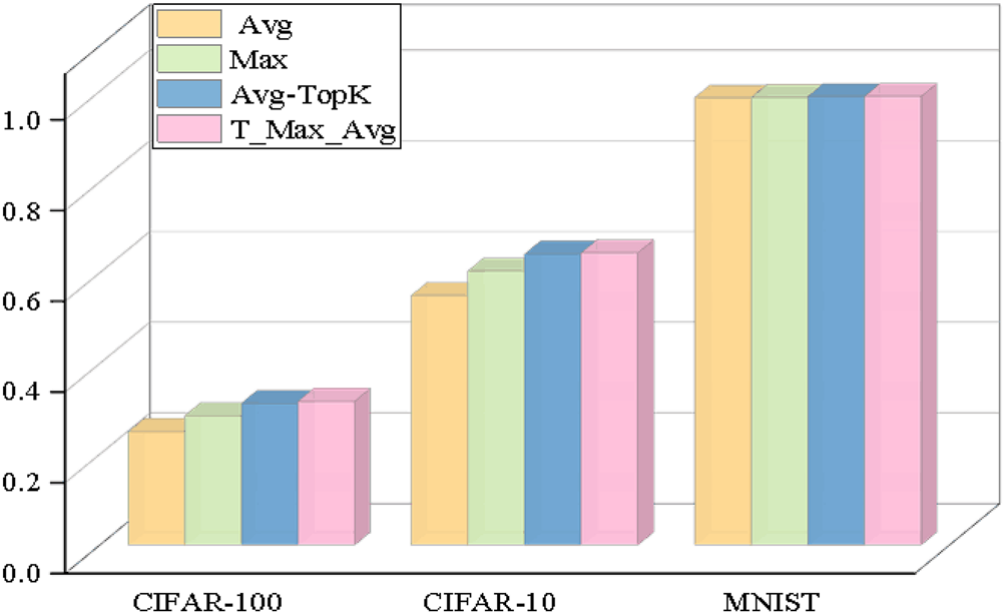
Success of pooling methods according to datasets.

Figure 13 illustrates the highest scoring outcomes obtained from the LeNet-5 architecture in experimental studies conducted on three distinct datasets, using various pooling methods.

**Table 8:** Experimental results with Pool-size = 4 and T = 0.7 on CIFAR-100 dataset.

CIFAR-100	Exp1 (%)	Exp2 (%)	Exp3 (%)	Exp4 (%)	Exp5 (%)	Exp6 (%)	Average (%)	Avg-TopK(Average) (%)
Avg	–	–	–	–	–	–	24.82	–
Max	–	–	–	–	–	–	27.67	–
T-Max-Avg2	29.58	28.64	28.76	29.47	28.92	28.13	28.92	29.21
T-Max-Avg3	31.16	30.51	30.63	30.07	29.2	30.48	30.34	29.43
T-Max-Avg4	30.5	30.41	29.79	30.89	31.33	30.56	30.58	30.42
T-Max-Avg5	30.95	29.8	31.09	31.23	31.21	31.47	30.96	30.25
T-Max-Avg6	32.98	32.3	31.36	31.42	31.7	30.93	31.78	31.25
T-Max-Avg7	32.27	30.77	30.48	31.39	31.15	30.55	31.1	30.83
T-Max-Avg8	32.23	31.24	30.92	29.05	31.43	31.79	31.11	30.97
T-Max-Avg9	30.2	30.31	30.81	30.67	30.52	31.27	30.63	30.99
T-Max-Avg10	30.74	30.84	30.42	29.51	30.16	31.75	30.57	30.47

**Table 9:** Experimental results with Pool-size = 4 and T = 0.7 on CIFAR-10 dataset.

CIFAR-10	Exp1 (%)	Exp2 (%)	Exp3 (%)	Exp4 (%)	Exp5 (%)	Exp6 (%)	Average (%)	Avg-TopK(Average) (%)
Avg	–	–	–	–	–	–	53.9	–
Max	–	–	–	–	–	–	60.1	–
T-Max-Avg2	61.12	61.98	62.11	61.24	61.91	62.28	61.77	61.83
T-Max-Avg3	63.15	62.74	63.95	62.84	63.55	63.21	63.24	62.98
T-Max-Avg4	62.83	62.79	62.87	63.47	61.44	62.15	62.59	63.16
T-Max-Avg5	62.94	64.13	62.96	63.53	62.49	64.25	63.38	62.54
T-Max-Avg6	64.33	64.57	64.61	63.92	65.17	63.94	64.42	64.14
T-Max-Avg7	63.16	63.54	62.05	62.56	63.09	63.95	63.06	63.16
T-Max-Avg8	62.93	61.55	62.93	63.48	62.29	63.14	62.72	62.2
T-Max-Avg9	61.6	62.5	62.59	62.79	60.81	62.72	62.17	62.73
T-Max-Avg10	62.69	60.87	61.35	62.15	60.23	61.4	61.45	61.02

**Table 10:** Experimental results with Pool-size = 4 and T = 0.8 on MNIST dataset.

MNIAT	Exp1 (%)	Exp2 (%)	Exp3 (%)	Exp4 (%)	Exp5 (%)	Exp6 (%)	Average (%)	Avg-TopK(Average) (%)
Avg	–	–	–	–	–	–	97.75	–
Max	–	–	–	–	–	–	97.76	–
T-Max-Avg2	97.82	97.44	98.17	97.94	97.88	97.89	97.86	98.17
T-Max-Avg3	98.02	97.75	98.06	98.18	98.28	98.19	98.08	98.28
T-Max-Avg4	98.19	97.95	98.26	98.18	98	98.22	98.13	98.05
T-Max-Avg5	98.36	98.32	98.14	98.46	98.24	98	98.25	98.25
T-Max-Avg6	97.85	98.63	98.12	98.07	98.21	98.24	98.19	98.1
T-Max-Avg7	98.37	98.22	98.22	98.07	98.5	98.24	98.27	98.18
T-Max-Avg8	98.24	98.11	98.36	98.08	98.23	97.72	98.12	98.05
T-Max-Avg9	98.32	98.01	98.12	98.24	98.24	98.09	98.17	98.14
T-Max-Avg10	98.23	98.32	98.24	98.14	98.47	98.32	98.29	98.06

**Table 11:** Experimental results with Pool-size = 4 and K = 6 on CIFAR-100 dataset.

CIFAR-100	Exp1 (%)	Exp2 (%)	Exp3 (%)	Exp4 (%)	Exp5 (%)	Exp6 (%)	Average (%)
T-Max-Avg(T = 0.1)	30.4	30.15	30.16	29.72	29.68	29.85	29.99
T-Max-Avg(T = 0.2)	30.35	28.89	30.44	29.08	30.2	29.69	29.78
T-Max-Avg(T = 0.3)	29.52	30.96	30.01	29.17	30.64	29.54	29.97
T-Max-Avg(T = 0.4)	29.92	30.52	31.01	30.75	30.76	31.32	30.71
T-Max-Avg(T = 0.5)	32.02	31.89	30.93	30.36	30.71	30.82	31.12
T-Max-Avg(T = 0.6)	31.3	30.22	31.04	30.08	30.47	31.27	30.73
T-Max-Avg(T = 0.7)	32.98	32.3	31.36	31.42	31.7	30.93	31.78
T-Max-Avg(T = 0.8)	30.55	30.72	31.62	32.99	30.84	30.47	31.2
T-Max-Avg(T = 0.9)	30.01	31.83	31.82	31.93	30.59	30.2	31.06

**Table 12:** Experimental results with Pool-size = 4 and K = 6 on CIFAR-10 dataset.

CIFAR-10	Exp1 (%)	Exp2 (%)	Exp3 (%)	Exp4 (%)	Exp5 (%)	Exp6 (%)	Average (%)
T-Max-Avg(T = 0.1)	63.19	63.79	60.48	63.6	62.33	61.17	62.43
T-Max-Avg(T = 0.2)	62.36	61.17	60.84	62.56	62.9	62.65	62.08
T-Max-Avg(T = 0.3)	61.69	63.02	63.8	63.57	62.37	62.81	62.88
T-Max-Avg(T = 0.4)	62.13	63.31	62.07	63.85	63.29	63.27	62.99
T-Max-Avg(T = 0.5)	61.53	64.13	64.11	63.53	64.01	63.58	63.48
T-Max-Avg(T = 0.6)	64.21	63.39	64.38	63.2	64.81	63.59	63.93
T-Max-Avg(T = 0.7)	64.33	64.57	64.61	63.92	65.17	63.94	64.42
T-Max-Avg(T = 0.8)	64.38	64.1	63.03	63.65	64.46	63.03	63.78
T-Max-Avg(T = 0.9)	63.75	63	64.38	63.97	63.52	63.15	63.63

**Table 13:** Experimental results with Pool-size = 2 and K = 2 on MNIST dataset.

MNIST	Exp1 (%)	Exp2 (%)	Exp3 (%)	Exp4 (%)	Exp5 (%)	Exp6 (%)	Average (%)
T-Max-Avg(T = 0.1)	98.73	98.55	98.67	98.78	98.63	98.39	98.64
T-Max-Avg(T = 0.2)	98.22	98.69	98.58	98.44	98.68	98.56	98.53
T-Max-Avg(T = 0.3)	98.26	98.51	98.68	98.4	98.55	98.64	98.51
T-Max-Avg(T = 0.4)	98.47	98.72	98.74	98.6	98.66	98.52	98.62
T-Max-Avg(T = 0.5)	98.65	98.79	98.78	98.84	98.58	98.59	98.7
T-Max-Avg(T = 0.6)	98.38	98.56	98.47	98.69	98.51	98.65	98.54
T-Max-Avg(T = 0.7)	98.71	98.66	98.69	98.62	98.65	98.64	98.66
T-Max-Avg(T = 0.8)	98.7	98.8	98.57	98.76	98.59	98.74	98.7
T-Max-Avg(T = 0.9)	98.5	98.64	98.34	98.71	98.54	98.62	98.56%

**Table 14:** Highest scoring performances of pooling methods on datasets.

Pool-size	2	3	4
CIFAR-100			
Avg Pooling	21%	25%	24.82%
Max Pooling	26.1%	28.5%	27.67%
Avg-TopK Pooling	26.63% (K = 2)	29.95% (K = 3)	31.25% (K = 6)
T-Max-Avg Pooling	28.03% (K = 1, T = 0.7)	30.1% (K = 3, T = 0.7)	31.7% (K = 6, T = 0.7)
CIFAR-10			
Avg Pooling	50%	55%	53.9%
Max Pooling	58.1%	60.4%	60.1%
Avg-TopK Pooling	59% (K = 2)	62.6% (K =3)	64.14% (K = 6)
T-Max-Avg Pooling	61.45% (K = 2, T = 0.7 )	63.83% (K = 3, T =0.7)	64.42% (K = 6, T = 0.7)
MNIST			
Avg Pooling	98.38%	98.57%	97.75%
Max Pooling	98.62%	98.63%	97.76%
Avg-TopK Pooling	98.63% (K = 3)	98.87% (K = 1)	98.28% (K = 3)
T-Max-Avg Pooling	98.73% (K = 2, T = 0.8)	98.88% (K = 3, T = 0.8)	98.29% (K = 10, T = 0.8)

From Figure 12, it can be seen that the T-Max-Avg method achieves comparable performance on the MNIST dataset with pool size of 2 and K value of 2 at both T = 0.5 and T = 0.8. Through further experimental comparisons, T = 0.8 is found to be a more ideal learning parameter, as shown in Tables 4, Tables 7, and Tables 10.

According to Table 14, our proposed T-Max-Avg method achieves the highest scores among all datasets.

- In color images, using the traditional pooling method with a pool size of 3 achieves the best score.
- In color images, using the T-Max-Avg method achieves the highest score when selecting a pooling size of 4 and a value of T equal to 0.7.
- The T-Max-Avg method demonstrates a more significant improvement on the CIFAR-10 dataset. With a pooling size of 3, K value of 3, and T equal to 0.7, it achieves a performance boost of 1.23% compared to the average top-K approach and a performance improvement of 3.43% compared to the average maximal pooling approach. It exhibits an improvement of 8.83% compared to the average pooling’s average optimal value performance.
- The T-Max-Avg method can further enhance the accuracy of the Avg-TopK approach on the CIFAR-10 dataset. With a pooling size of 2, K value of 1, and T equal to 0.7, it achieves a performance improvement of 1.4% compared to the average top-K approach and a performance improvement of 1.93% compared to the average maximal pooling approach. It shows an improvement of 7.03% compared to the average pooling’s average optimal value performance.
- The T-Max-Avg method performs more ideally and successfully compared to the Avg-TopK method on the MNIST dataset. With a pool size of 2, K value of 2, and T value of 0.8, it achieves a 0.1% improvement in average top-K performance compared to Avg-TopK. It also outperforms maximum pooling with a 0.11% improvement in average top-K performance. Compared to average pooling, it achieves a 0.35% improvement in average top-K performance.

## Expansion experiment

### Experiment description

To investigate the effectiveness of T-Max-Avg pooling technique in transfer learning models, we conducted a series of experiments. These experiments used the CIFAR-10 dataset and applied the proposed pooling method to the pooling layers of traditional transfer learning models. We did not fine-tune the models but kept the parameters of the pooling layers unchanged to ensure that the transfer learning models had the same number of parameters. As there are no pooling layers in models such as MobileNet^31^, MobileNetV2, and MobileNetV3, we did not study these models. Instead, we selected , VGG19^32^, ResNet50^33^, and ChestX^34^ models for experimentation.

**Figure 14:**
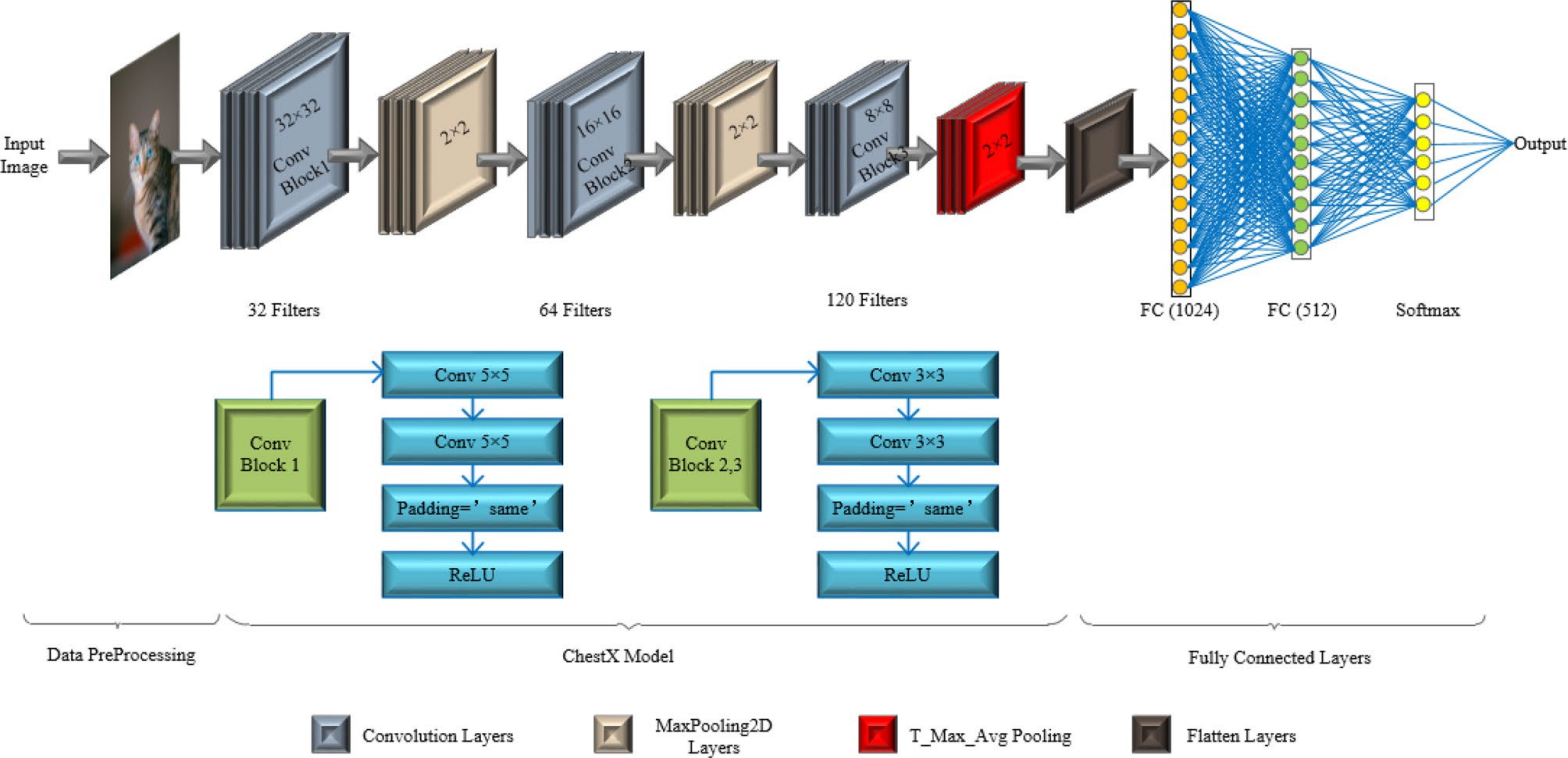
ChestX(T-Max-Avg) Model architecture.

In the experiments on VGG19 and ResNet50 transfer models, we set the K value of the T-Max-Avg pooling layer to 3. We found that the VGG19 model was unable to learn from the CIFAR-10 dataset. The ChestX model is a high-performance, low-latency, and high-accuracy convolutional neural network model proposed by Md. Nahiduzzaman et al. To investigate if the T-Max-Avg pooling method improves the accuracy of the ChestX model, we replaced the last max pooling layer with a T-Max-Avg pooling layer, setting the pool size to 2 and K value to 2, while keeping the parameters of the ChestX model unchanged. The model structure can be referred to Figure 14 and Table 15.

**Table 15:** ChestX(T-Max-Avg) network architecture.

Layer	Layer Type	Size of Feature Map	Kernel size	Param	Activation Functions
Input Layer	Image	32$\times$32$\times$3	–	–	–
Conv1-1 Layer	Convolution	32$\times$32$\times$32	5$\times$5	2432	ReLU
Conv1-2 Layer	Convolution	32$\times$32$\times$32	5$\times$5	25,632	ReLU
Pool1 Layer	MaxPooling	16$\times$16$\times$32	2$\times$2	0	–
Conv2-1 Layer	Convolution	16$\times$16$\times$64	3$\times$3	18,496	ReLU
Conv2-2 Layer	Convolution	16$\times$16$\times$64	3$\times$3	36,928	ReLU
Pool2 Layer	MaxPooling	8$\times$8$\times$64	2$\times$2	0	–
Conv3-1 Layer	Convolution	8$\times$8$\times$128	3$\times$3	73,856	ReLU
Conv3-2 Layer	Convolution	8$\times$8$\times$128	3$\times$3	147,584	ReLU
Pool3 Layer	T-Max-Avg	4$\times$4$\times$128	(2,2,0.7)	0	–
dropout1	Dropout	4$\times$4$\times$128	–	0	ReLU
flatten	Flatten	2048	–	0	ReLU
FC1 Layer	Full Connected	1024	–	2,098,176	ReLU
dropout2	Dropout	1024	–	0	ReLU
FC2 Layer	Full Connected	512	–	524,800	ReLU
dropout3	Dropout	512	–	0	ReLU
FC3 Layer	Full Connected	10	–	5,130	Softmax

**Figure 15:**
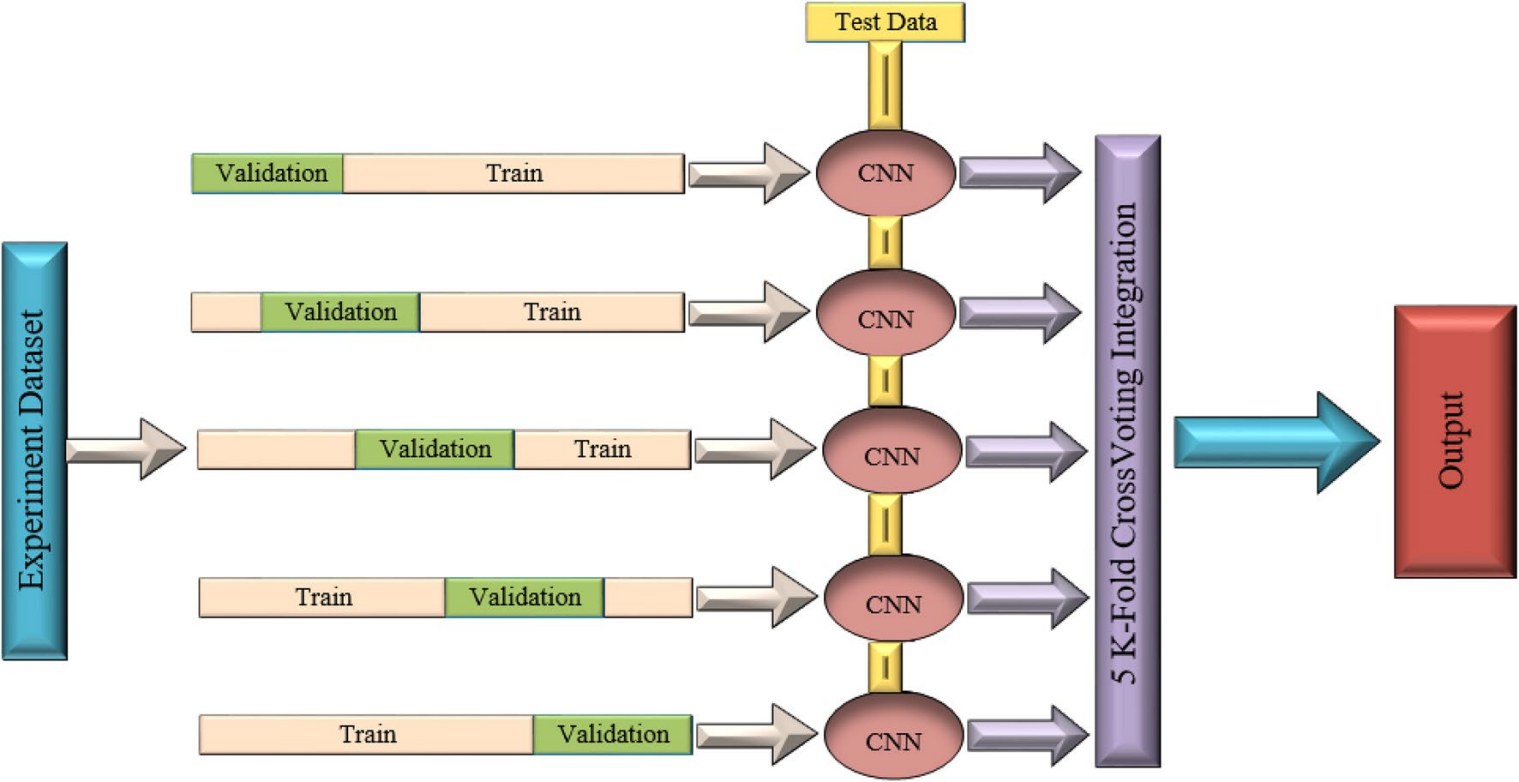
5 K-Fold cross validation ensemble model architecture.

To further improve the classification accuracy on the CIFAR-10 dataset, we employed cross-validation ensemble for further enhancement, based on the experimental results and experience of J. D. Domingo et al.^35^. We adopted a five-fold cross-validation ensemble, and the model structure is shown in Figure 15. For detailed experimental results, please refer to Table 16. The formula for five-fold cross-validation ensemble is defined as follows:

5 $$\begin{aligned} S(x)=c_{\arg g_{j} \max \sum _{i=1}^{k} w_{i} \cdot p_{i j}(x)} \end{aligned}$$

K represents the number of classifiers, each associated with its respective validation slot. x is an input sample, and pi(x) is a vector of output probabilities given by classifier i. This vector consists of probabilities pij(x), where classifier i represents the probability of the sample x belonging to class j. c represents the vector of labels. In Eq. (5), soft voting^36^ is obtained by accumulating the output probabilities for each class j. wi represents the weight associated with each classifier i, which in our example is 1/k. The argmax function returns the position of the class with the highest cumulative probability.

Hard voting^36^ requires binarizing the probabilities, where the class with the highest probability is set to 1 and the rest are set to 0. This step can be implemented using Eq. (6).

6 $$\begin{aligned} b_{i j}(x)=\left\{ \begin{array}{lr}1 &{} \text{ if } p_{i j}(x)=\max \left( p_{i}(x)\right) \\ 0 &{} \text{ otherwise } \end{array}\right. \end{aligned}$$

In Eq. (7), the output is obtained by accumulating the binary values of each class j. As in soft voting, wi is 1/k in our case.

7 $$\begin{aligned} H(x)=c_{a \arg g_{j} \max \sum _{i=1}^{k} w_{i} \cdot b_{i j}(x)} \end{aligned}$$

In this experiment, we set the batch-size to 128 and the number of epochs to 50 to facilitate faster convergence of the experimental results.In the ChestX model, we set the learning parameter T of the T-Max-Avg pooling method to 0.7.

### Performance matrix for classification

In this study, we have used performance metrics such as accuracy, precision, recall, F1-score, and area under the curve (AUC) to compare the results. After training the ResNet50 and ChestX models, we calculated their performance on the testing dataset using AUC. High accuracy can be achieved when the model has high precision and correctness^37^. The accuracy is determined using Eq. (8). Precision is defined as the fraction of relevant samples among the recovered samples, and it is obtained using Eq. (9)^38^. Recall, on the other hand, is defined as the fraction of total relevant samples that are correctly recovered and can be calculated using Eq. (10)^38^. The F1-score measures the harmonic mean and is given by Eq. (11)^39^.

8 $$\begin{aligned} Accuracy=\frac{TP+TN}{TP+TN+FP+FN} \end{aligned}$$

9 $$\begin{aligned} Precision=\frac{TP}{TP+FP} \end{aligned}$$

10 $$\begin{aligned} Recall=\frac{TP}{TP+FN} \end{aligned}$$

11 $$\begin{aligned} F-score=2\times \frac{Precision\times Recall}{Precision+Recall} \end{aligned}$$

In the formulas, TP refers to True Positive, which represents the number of samples correctly classified as positive. TN stands for True Negative, which represents the number of samples correctly classified as negative. FP represents False Positive, indicating the number of samples that are actually negative but incorrectly classified as positive. FN represents False Negative, representing the number of samples that are actually positive but incorrectly classified as negative. These terms are commonly used in binary classification tasks to evaluate model performance and calculate metrics such as accuracy, precision, recall, and F1-score.

### Experiment result

**Figure 16:**
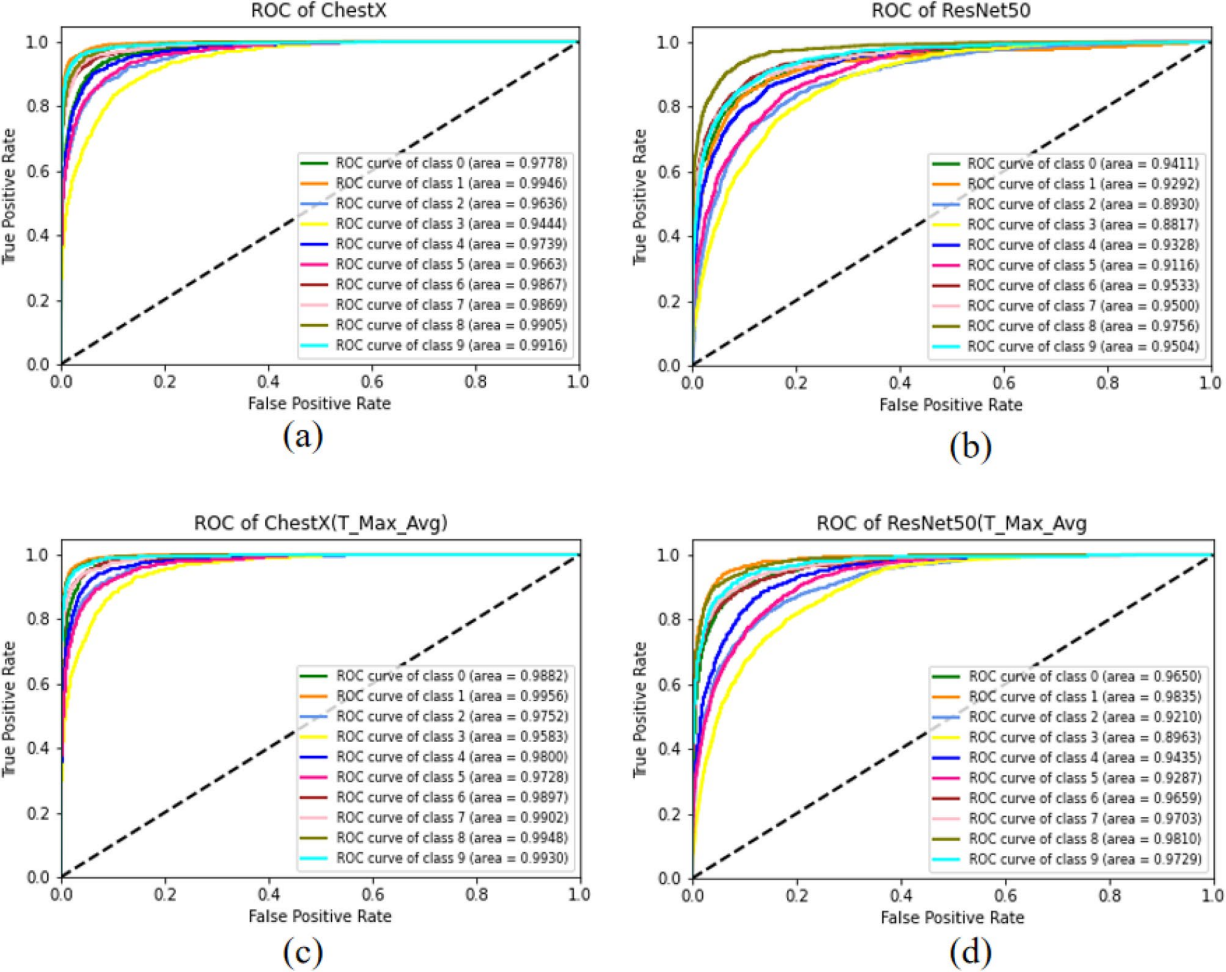
Visualizing the ROC curve for the model, (**a**) ChestX, (**b**) ResNet50 ,(**c**) ChestX (T-Max-Avg), (**d**) ResNet50 (T-Max-Avg).

**Figure 17:**
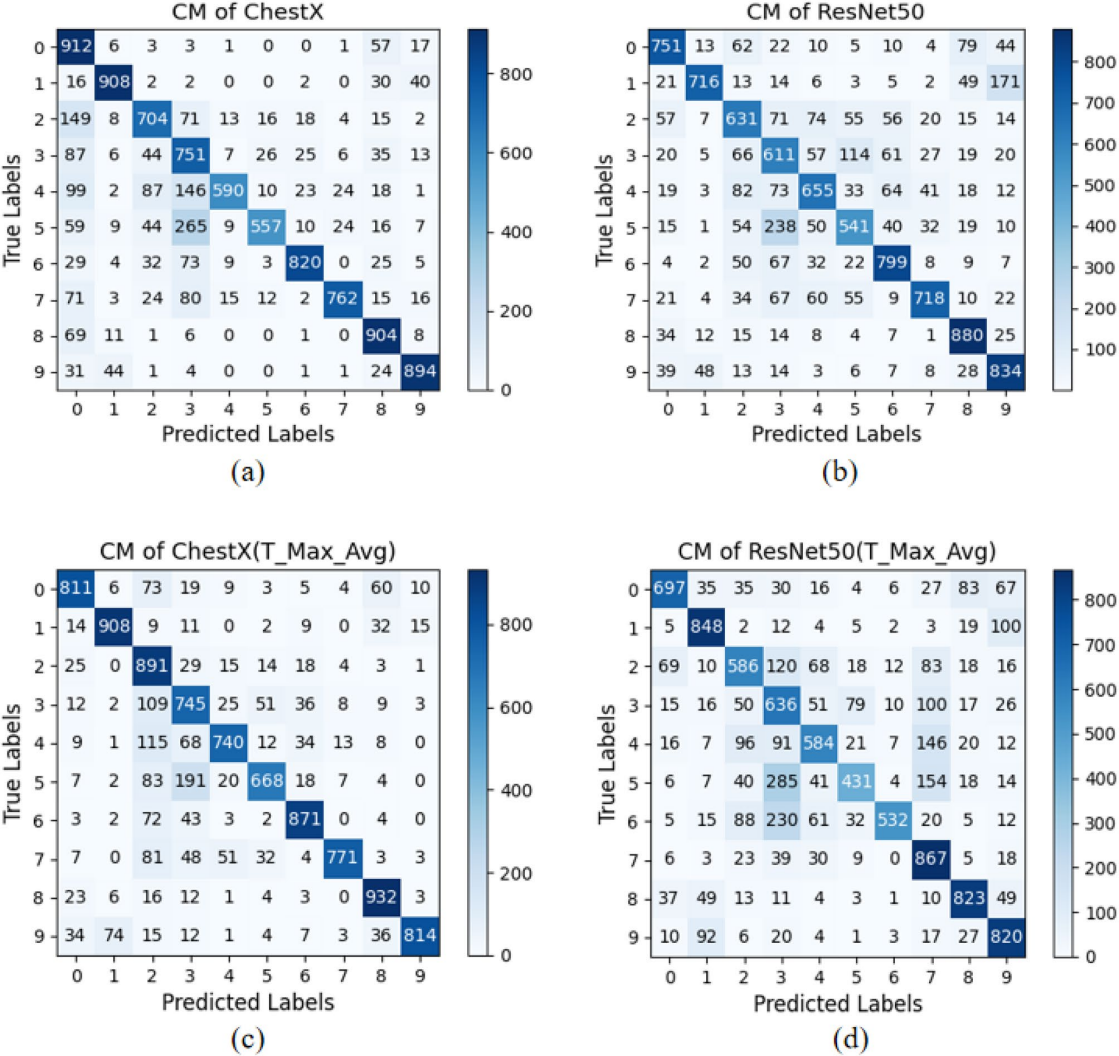
Visualizing the confusion matrix for the model, (**a**) ChestX, (**b**) ResNet50 ,(**c**) ChestX (T-Max-Avg), (**d**) ResNet50 (T-Max-Avg).

In this study, we utilized the CIFAR-10 dataset to train and test the ChestX, ResNet50, and DenseNet121 models. Subsequently, we applied our proposed T-Max-Avg pooling method to each model and conducted comparative experiments. The results of the experimental comparison are presented in Table 16 and Table 17. Following that, we visualized the experimental results by plotting ROC curves and confusion matrices for the ChestX, ResNet50, ChestX(T-Max-Avg) and ResNet50(T-Max-Avg) models. These visualizations are shown in Figure 16 and Figure 17, respectively.

To validate the effectiveness of our proposed T-Max-Avg method, we replaced the original max pooling layer in the above models with the T-Max-Avg pooling method, and used it only once. Experimental results have shown that the T-Max-Avg pooling method has achieved significant success on these three models. In the ChestX model, besides the similar accuracy and recall rates, the T-Max-Avg pooling method has shown better performance in other metrics compared to the original model. In the ResNet50 model, except for a lower precision in the 5-fold cross-validation ensemble, there has been an improvement of nearly 2.5% in other evaluation metrics.

**Table 16:** Comparison of experimental results between ChestX and ChestX (T-Max-Avg).

	Accuracy (%)	Precision (%)	Recall (%)	F1-score (%)
	83.26	83.71	83.26	83.19
	81.33	82.05	81.33	81.26
ChestX	83.08	83.85	83.08	83.12
	82.91	83.76	82.91	82.98
	78.02	81.2	78.02	78.22
Average	81.72	82.91	81.72	81.75
5-KFold Hard Voting Ensemble	85.58	86.32	85.58	85.66
5-KFold Soft Voting Ensemble	86.32	86.87	86.32	86.35
	81.14	84.47	81.14	81.89
	82.73	82.73	82.73	82.37
ChestX(T-Max-Avg(2, 2, 0.7))	78.53	81.71	78.53	78.78
	84.47	84.58	84.47	84.41
	81.51	83.37	81.51	81.81
Average	81.68	83.37	81.68	81.85
5-KFold Hard Voting Ensemble	86.31	86.72	86.31	86.44
5-KFold Soft Voting Ensemble	86.98	87.19	86.98	87.05

**Table 17:** Comparison of experimental results between ResNet50 and ResNet50 (T-Max-Avg).

	Accuracy	Precision (%)	Recall (%)	F1-score (%)
	71.36	72.02	71.36	71.39
	56.8	61.45	56.8	56.03
ResNet50	55.4	64.56	55.4	56.54
	70	71.05	70	70.05
	68.1	70.07	68.1	68.18
Average	64.33	67.83	64.33	64.44
5-KFold hard voting ensemble	73.81	75.48	73.81	74.08
5-KFold soft voting ensemble	75.26	76.38	75.26	75.4
	68.24	70.83	68.24	68.11
	66.46	68.91	66.46	66
ResNet50(T-Max-Avg(3, 3, 0.6))	62.91	66.78	62.91	63.02
	70	71.92	70	70.05
	66.83	66.71	66.83	66.51
Average	66.89	69.03	66.89	66.74
5-KFold hard voting ensemble	74.48	75.11	74.48	74.51
5-KFold Soft voting ensemble	75.47	75.87	75.47	75.4

## Conclusion and future work

In the architecture of convolutional neural networks (CNNs), the pooling layer is used to reduce the dimensions of feature maps. However, traditional pooling methods often result in data loss, although they can decrease file size. This study proposes a novel pooling method aimed at overcoming the potential information loss incurred during traditional pooling processes. We extensively compare this new pooling method with Avg-TopK, max, and average pooling methods on three different datasets.

The research conducted using the LeNet-5 convolutional neural network model on three different datasets has shown that the max pooling method is more effective than the average pooling method. We have observed that our proposed T-Max-Avg method outperforms max pooling and average pooling, and further improves the accuracy of the Avg-TopK pooling method. It is more stable than these three methods and demonstrates a higher compatibility rate.

In the proposed T-Max-Avg method, for color images, selecting a pool-size of 4, K value of 6, and T value of 0.7 resulted in the highest score. For grayscale images, selecting a pool-size of 3, K value of 3, and T value of 0.8 yielded the highest score. The appropriate learning parameter T may vary depending on the dataset. To determine the optimal pool size, K value, and T value for the dataset implementing this method, a larger range of experimental research is required.

When applied to transfer learning models, it has been observed that the T-Max-Avg method achieves more successful results on ChestX, ResNet50, and DenseNet121 models compared to traditional pooling methods. Afterwards, by means of cross-ensemble techniques, the model is further improved to achieve higher accuracy and minimize errors.

The T-Max-Avg method is a combination of the Avg-TopK pooling method and the maximum pooling method. Its design aims to eliminate the drawbacks of traditional pooling methods. In experimental studies, the T-Max-Avg method has been found to be more effective than using the Avg-TopK method alone. It provides a simple, fast, and convenient new pooling method, addressing the preference for traditional methods in deep learning model design.

Our future research will focus on reducing the time complexity of the T– method while maintaining its accuracy. We aim to achieve a win-win effect in terms of both precision and time.

## Data Availability

All data generated or analysed during this study are included in this published article.
